# A 3D subject-specific model of the spinal subarachnoid space with anatomically realistic ventral and dorsal spinal cord nerve rootlets

**DOI:** 10.1186/s12987-017-0085-y

**Published:** 2017-12-19

**Authors:** Lucas R. Sass, Mohammadreza Khani, Gabryel Connely Natividad, R. Shane Tubbs, Olivier Baledent, Bryn A. Martin

**Affiliations:** 1 Neurophysiological Imaging and Modeling Laboratory, University of Idaho, 875 Perimeter Dr. MC1122, Moscow, ID 83844-1122 USA; 2 Bioflow Image, Service de Biophysique et de Traitement de l’Image médicale, Bâtiment des écoles, CHU Nord Amiens-Picardie, Place Victor Pauchet, 80054 Amiens Cedex 1, France; 3 Seattle Science Foundation, 200 2nd Ave N, Seattle, WA 98109 USA; 4 Department of Biological Engineering, University of Idaho, 875 Perimeter Dr. MC0904, Moscow, ID 83844-0904 USA

**Keywords:** Spinal subarachnoid space, Intrathecal drug delivery, 3D reconstruction, Cerebrospinal fluid, Spinal cord, Dura mater, Nerve roots, Spinal cord injury, Neurapheresis, Cerebrospinal fluid hypothermia

## Abstract

**Background:**

The spinal subarachnoid space (SSS) has a complex 3D fluid-filled geometry with multiple levels of anatomic complexity, the most salient features being the spinal cord and dorsal and ventral nerve rootlets. An accurate anthropomorphic representation of these features is needed for development of in vitro and numerical models of cerebrospinal fluid (CSF) dynamics that can be used to inform and optimize CSF-based therapeutics.

**Methods:**

A subject-specific 3D model of the SSS was constructed based on high-resolution anatomic MRI. An expert operator completed manual segmentation of the CSF space with detailed consideration of the anatomy. 31 pairs of semi-idealized dorsal and ventral nerve rootlets (NR) were added to the model based on anatomic reference to the magnetic resonance (MR) imaging and cadaveric measurements in the literature. Key design criteria for each NR pair included the radicular line, descending angle, number of NR, attachment location along the spinal cord and exit through the dura mater. Model simplification and smoothing was performed to produce a final model with minimum vertices while maintaining minimum error between the original segmentation and final design. Final model geometry and hydrodynamics were characterized in terms of axial distribution of Reynolds number, Womersley number, hydraulic diameter, cross-sectional area and perimeter.

**Results:**

The final model had a total of 139,901 vertices with a total CSF volume within the SSS of 97.3 cm^3^. Volume of the dura mater, spinal cord and NR was 123.1, 19.9 and 5.8 cm^3^. Surface area of these features was 318.52, 112.2 and 232.1 cm^2^ respectively. Maximum Reynolds number was 174.9 and average Womersley number was 9.6, likely indicating presence of a laminar inertia-dominated oscillatory CSF flow field.

**Conclusions:**

This study details an anatomically realistic anthropomorphic 3D model of the SSS based on high-resolution MR imaging of a healthy human adult female. The model is provided for re-use under the Creative Commons Attribution-ShareAlike 4.0 International license (CC BY-SA 4.0) and can be used as a tool for development of in vitro and numerical models of CSF dynamics for design and optimization of intrathecal therapeutics.

**Electronic supplementary material:**

The online version of this article (10.1186/s12987-017-0085-y) contains supplementary material, which is available to authorized users.

## Background

Detailed analysis of cerebrospinal fluid (CSF) dynamics is thought to be of importance to help understand diseases of the central nervous system such as Chiari malformation [1], hydrocephalus [2, 3] and intracranial hypertension [4]. CSF therapeutic interventions have also been investigated such as intrathecal drug delivery [5], CSF filtration or “neurapheresis” (also previously termed liquorpheresis) [6, 7] and CSF hypothermia (cooling) treatment [8]. The exact relation, if any, of CSF dynamics to these disorders and treatments is under investigation. There are many opportunities for researchers to make a contribution to the field.

A significant contribution to our understanding of CSF dynamics has been made by the use of computational fluid dynamics (CFD) modeling; an engineering technique that allows detailed analysis of the CSF flow field that is not possible by MRI measurements or invasive means. In addition, CFD allows for variational analysis, where specific parameters in the model can be altered to understand their distinct contribution. Major CFD-based contributions to our knowledge of CSF physiology have been made in the areas of CSF ventricular dynamics [9], drug transport [10, 11], filtration [12], alterations in brain pathologies [13–15], spinal cord pathology [16] and wave mechanics [17, 18].

Computational fluid dynamics modeling relies on accurate representation of boundary conditions that are difficult to define because of the intricate spinal subarachnoid space (SSS) geometry, complex CSF flow field and lack of material property information about the central nervous system tissues. Each CFD modeling approach has necessitated varying degrees of boundary condition simplification with respect to anatomy and physiology. When considering anatomy, CFD models that attempt to accurately imitate the spinal geometry are generally built from subject-specific MRI scans. However, even for experts in spinal neuroanatomy, magnetic resonance (MR) imaging resolution and artifacts make subject-specific anatomical reconstruction of the SSS difficult, particularly for engineers who often have limited anatomical knowledge. Herein, we provide to the research community an open-source subject-specific 3D model of the complete SSS with idealized spinal cord nerve rootlets (NR) licensed under the Creative Commons Attribution-ShareAlike 4.0 International license (CC BY-SA 4.0). This also includes the in vivo measured CSF flow waveforms along the spine. The open-source model can allow multiple researchers a tool to investigate and compare results for CSF dynamics related phenomena and technologies such as pharmacokinetics of intrathecal drug distribution, neurapheresis and hypothermia.

## Methods

### Subject selection

A single, representative healthy, 23-year-old, female Caucasian subject was enrolled in this study. The subject had no previous history of neurological or cardiovascular disorders.

### MRI CSF flow measurement protocol

All MRI measurements were obtained with a General Electric 3T scanner (Signa HDxt, software 15.0_M4_0910.a). CSF flow data were collected at three vertebral levels, C2–C3, C7–T1 and T10–T11, using phase-contrast MRI with retrospective electrocardiogram (ECG) gating and 32 cardiac phases [14]. Each slice had a thickness of 5.0 mm and an in-plane resolution of 0.54 × 0.54 mm. Orientation of the slice was made perpendicular to the CSF flow direction and positioned vertically by intersection with a vertebral disk (i.e. C2–C3). A flip angle, TR, TE and VENC was used with a value of 25°, 13.4, 8.26 and 8 cm/s respectively. Detailed information on imaging parameters is provided by Baledent et al. [19].

### CSF flow quantification

Oscillatory cardiac-related CSF flow was quantified for the axial locations located at the vertebral disk at the C2–C3, C7–T1 and T10–T11 vertebral levels. As detailed in our previous studies [14, 20], Matlab was used to compute the CSF flow waveform, *Q*_(*t*)_, based on integration of the pixel velocities with *Q*(*t*) = ∑*A*_*pixel*_ [*V*_*pixel*_(*t*)], where *A*_*pixel*_ is the area of one MRI pixel, *V*_*pixel*_ is the velocity for the corresponding pixel, and *Q*_(*t*)_ is the summation of the flow for each pixel of interest. A smooth distribution of CSF flow along the spine was achieved by interpolating CSF flow between each axial measurement location [21]. Similar to previous studies, the diastolic CSF flow cycle phase was extended in cases when necessary [22]. For correcting eddy current offsets, the cyclic net CSF flow was offset to produce zero net flow over a complete flow cycle [14].

### MRI CSF space geometry protocol

To collect geometric measurements with improved CSF signal, 3D fast imaging employing steady state acquisition (3D FIESTA) was used, and acquisitions were realized with free breathing. The coils used were the HD Neck-Spine Array with 16 Channels for the spine and the 29 element phased array for the upper-neck. Images were collected in three volumes, from the top of the brain to C7, from C5 to T9, and from T9 to S5, with each section containing 140, 104 and 104 sagittal T2-weighted images respectively. The field of view (FOV) size was 30 cm × 30 cm × 7 cm for the craniocervical volume, and 30 cm × 30 cm × 5.25 cm for both the thoracic and lumbosacral volumes. In-plane voxel spacing was 0.547 × 0.547 mm and slice thickness was 1 mm with slice spacing set at 0.499 mm. Echo times (TE) were 1.944, 2.112, 2.100 and repetition times (TR) were 5.348, 5.762, 5.708 for the craniocervical, thoracic, and lumbosacral volumes respectively. Total imaging time for the three levels was ~ 45 min.

### CSF space segmentation

**Fig. 1:**
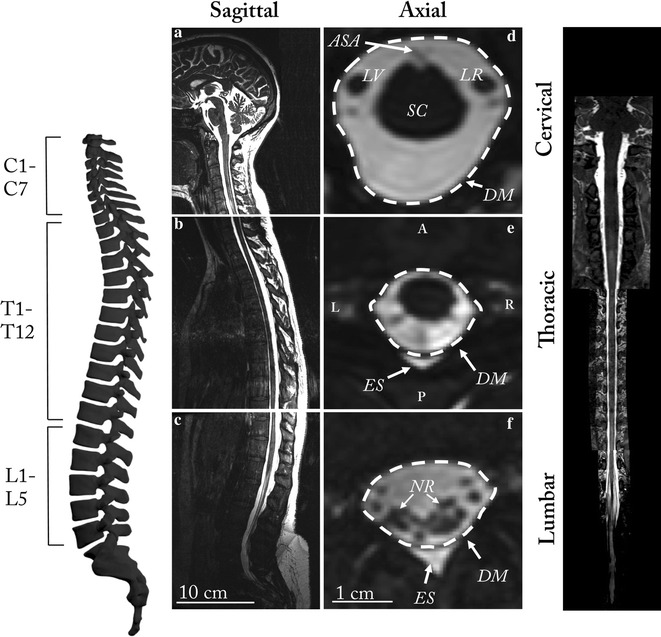
T2-weighted MRI data were collected as three volumes, **a** craniocervical, **b** thoracic, **c** Lumbosacral. A variety of artifacts exist in and around the SSS, **d**–**f** including the anterior spinal artery (ASA), left and right vertebral arteries (LV and LR), epidural space (ES), dura mater (DM), spinal cord (SC), and dorsal and ventral nerve rootlets (NR) in particular near the cauda equina. Note: the 3D geometry provided in this manuscript only includes the CSF within the spine below the foramen magnum (*L* left, *R* right, *A* anterior, *P* posterior)

The open-source program, ITK-SNAP (Version 3.4.0, University of Pennsylvania, USA) [23], was used to segment the MRI data. Similar to our previous work [24], the cervical, thoracic and lumbar MR image sets were manually segmented in the axial orientation using the semi-automatic contrast-based segmentation tool. The segmented region extended from the foramen magnum to the end of the dural sac. One expert operator completed the segmentation, as our previous study showed strong inter-operator reliability of SSS geometric parameters [24]. A second expert operator reviewed the images to confirm region selection, and in areas of disagreement, discussed in detail with respect to the anatomy. Hyperintensities in the T2-weighted image sets near the epidural space were excluded from the model segmentation **(**Fig. 1). MRI data were not collected in high-resolution for the entire brain, and thus the cortical and ventricular CSF spaces were not included in the model. After completion, each segmentation was exported as an .STL file with Gaussian smoothing option applied (standard deviation = 0.80 and maximum approximation error = 0.03).

### Model alignment

The open source program, Blender (Version 2.77a, Amsterdam, Netherlands), was used for the majority of mesh modifications and all modeling operations in this study. After segmentation, the .STL files generated were imported into Blender. Because of the global reference coordinate set by the MRI, segmentations generated from different image series were automatically registered. However, 3D rigid body translation (~ 5 mm maximum) was required to align each model section due to a small degree of subject movement between the MR image acquisitions. These translations were performed based on a visual best fit.

### Geometry remeshing and smoothing

**Fig. 2:**
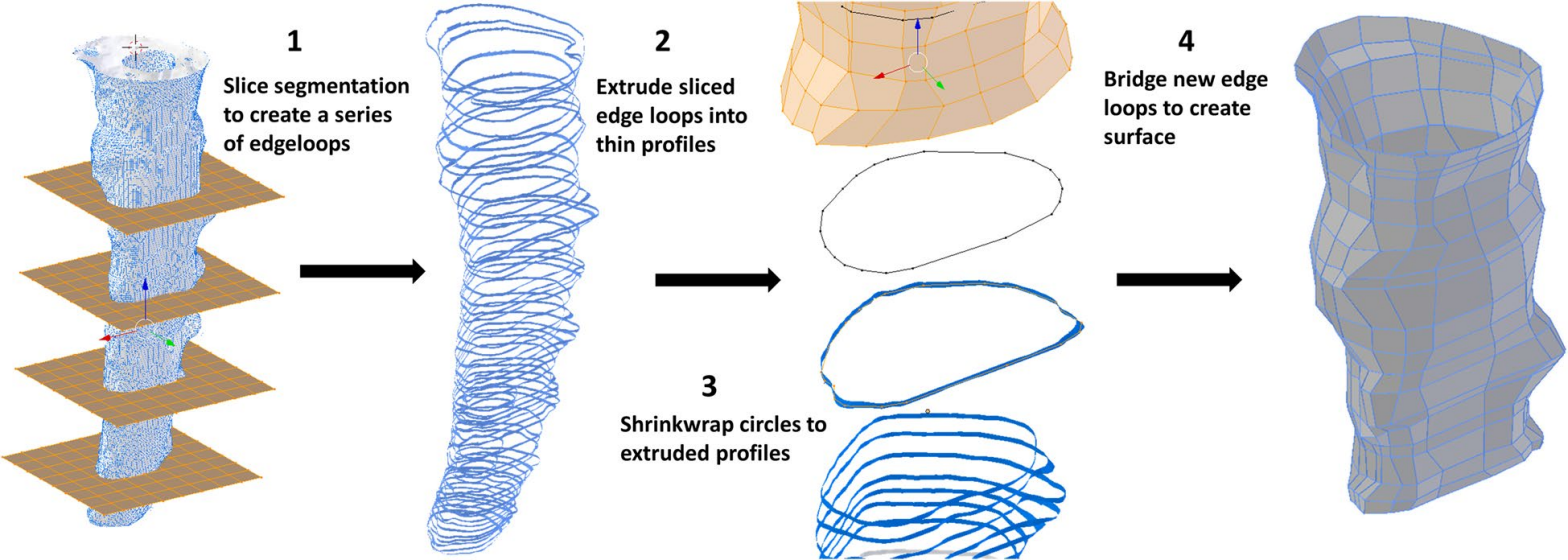
Geometric mesh optimization was performed to produce a simplified quadrilateral mesh from the original segmentation mesh

The following operations were completed to create a lowest-resolution semi-regular surface mesh of the spinal cord and dura while maintaining an accurate representation of the original geometry. After alignment, the triangulated .STL segmentations were converted to quadrilateral meshes using the automatic conversion tool “tris to quads” in Blender. The spinal cord and dural surfaces were separated, and an array of planes was placed along the entire spinal segmentation at a roughly orthogonal orientation to the spinal trajectory. Vertical spacing of these planes was determined by choosing an inter-plane interval (~ 5 mm) that preserved surface contours; this required a minimum of three planes to preserve a change in surface concavity. The circumferential contour of the spinal cord and dura was obtained at each plane using the “intersect (knife)” operation in Blender. The original geometry was then removed. Each surface contour was then vertically extruded ~ 1 mm. Simple circle meshes were place at each contour using the “add circle” command, the “shrink wrap” modifier was then used to form these circles around each profile. The number of vertices in the circles wrapped to the dural and spinal cord profiles was specified to be 55 and 32 respectively. These parameters were determined based on visual inspection of the shrink-wrap fit at the largest profile diameter located at the foramen magnum. Manual adjustment of individual vertices was made to preserve a uniform vertex distribution and surface contour at each slice. To create a continuous quadrilateral mesh of both the spinal cord and dura, the “bridge edge loops” command was used between adjacent contours (Fig. 2).

**Fig. 3:**
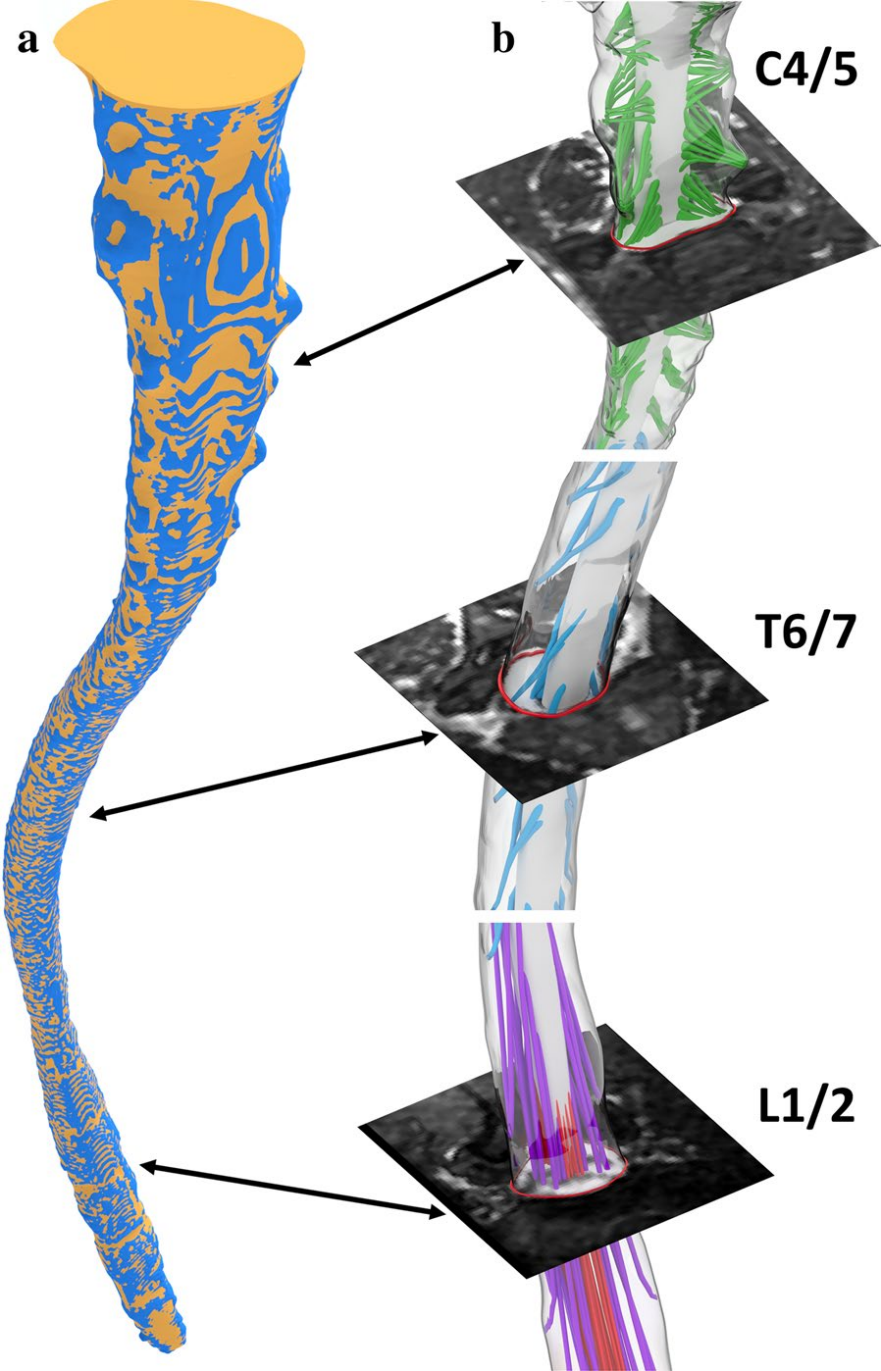
**a** The final dural and spinal cord surfaces (yellow) were visually compared to their respective segmentations (blue) through an overlay to determine the quality of the reconstruction. Manual sculpting was used to improve areas where there was surface bias. **b** For comparison, the final model is overlaid on representative axial MRI slices at three axial locations, C4/5, T6/7 and L1/2

Manual adjustments were then made by sculpting the remeshed surfaces within the “sculpt mode” workspace in Blender to produce ~ 50% visual interference with the original segmentation surface (Fig. 3). To further improve surface accuracy, a combination of a shrink-wrap and “smooth” modifiers were used simultaneously. Importantly, the “keep above surface” option and “offset” options on the shrink-wrap modifier were used. The values for shrink-wrap offset and smoothing factor in their respective modifier menus must be determined by a trial and error method for each unique mesh until the desired smoothness is justified with overall volume. In this study, values of 0.04 and 0.900 were used for offset and smoothing factor respectively.

### Nerve root modeling

**Fig. 4:**
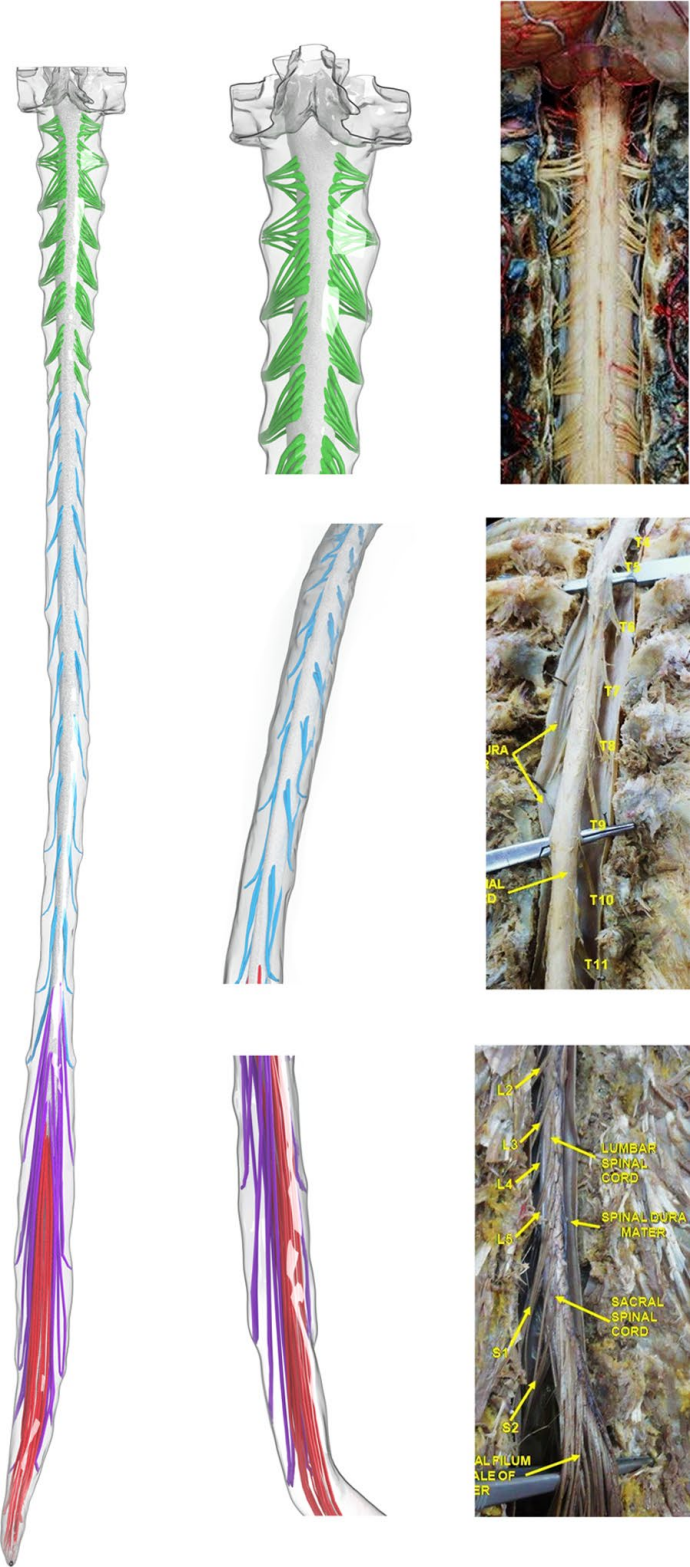
Complete spinal geometry showing detail in the cervical (green), thoracic (blue), lumbar (violet), and sacral (red) regions compared to anatomic imagery of respective locations [84–86]. Note: all model calculations are made for SSS region located below the foramen magnum only (picture shows part of foramen magnum for illustration of connection to brain)

The 31 NR pairs, starting from the craniocervical junction, were modeled using the following methodology. For each rootlet, a “circle” mesh was extruded from the SC junction to the dural exit location in Blender. The curvature, radicular line (RL) and descending angle (DA) for each rootlet were determined based on the subject specific segmentation, average cadaveric measurements from the literature and anatomic reference imagery [25–28] (Fig. 4). The exact method varied by location due to variations in the completeness of the data types; these differences are described below. Note: the 31st nerve root, or coccygeal nerve did not bifurcate into a nerve root pair until after leaving the intrathecal CSF space.

In the left side of the cervical spine, segmentations of the NR were possible to obtain directly from the anatomic MR imaging. These were imported and aligned with the existing model in Blender. A “circle” mesh was extruded along each segmented path and the diameter of this circle was defined as the average NR diameter or thickness from cadaveric measurements for each location. Additionally, in the cervical spine the spinal entry point of each rootlet cylinder was scaled in the cranial direction (~ 150%) along the spinal cord to create a blended transition. Finally, cervical rootlets were mirrored left to right and small adjustments were made to fit them to the correct exit points on the right side of the dura. Mirroring was applied as the NR intersection location at the spinal cord and dura was nearly identical for the left and right side NR.

In the thoracic spine, segmentations were only able to inform NR entry and exit points, and by extension, DA. It is possible that NR points in the thoracic spine were difficult to visualize within this region due to image blurring stemming from respiratory-related tissue motion. NR morphology in the thoracic spine is a steeply descending and tightly packed bundle. Therefore, to reduce unnecessary mesh complexity, a standard NR set was developed as a simplified cylinder with a diameter based on the average NR bundle size in the thoracic region. In addition to this main cylinder, a secondary cylinder was incorporated at the SC entry point to more closely imitate NR branching near the spinal cord. This cylinder extends from just below the primary rootlet entry point to a location approximately one-third of way along the primary rootlet; overall a steeply descending deltoid morphology is created. As in the cervical spine, a blended transition was created at the SC entry point for each NR. This standard NR set was mirrored left to right of the SC and duplicated along the SC for the entire thoracic region.

In the lumbosacral spine, the NR form the cauda equina. High MR image contrast made complete segmentations of this region possible and NR modeling was completed as in the cervical spine. NR were again simplified as a single cylinder of average diameter. Because of this, RLs for this region were not possible to define.

### Geometric analysis

Geometric parameters were calculated along the complete spinal mesh at 1 mm intervals [21]. SSS cross-sectional area, *A*_*cs*_ = *A*_*d*_ − *A*_*c*_ − *A*_*nr*_, was determined based on cross-sectional area of the NR (*A*_*nr*_), SC (*A*_*c*_) and dura (*A*_*d*_). Hydraulic diameter for internal flow within a tube, *D*_*H*_ = 4*A*_*cs*_/*P*_*cs*_, was determined based on the cross-sectional area and wetted perimeter, *P*_*cs*_ = *P*_*d*_ + *P*_*c*_ + *P*_*nr*_. Wetted perimeter was computed as the sum of the NR (*P*_*nr*_), SC (*P*_*c*_) and dura (*P*_*d*_) perimeters. Each of these parameters was calculated within a user defined function compiled in ANSYS FLUENT (Ver. 18.1, ANSYS inc, Canonsburg, PA). Note, for geometric analysis, the coccygeal nerve (spinal nerve) was considered to be a part of the spinal cord. 

### Hydrodynamic analysis

The hydrodynamic environment at 1 mm slice intervals along the entire spine was assessed by Reynolds number based on peak flow rate, $\text{Re} = \frac{{Q_{sys} D_{H} }}{{\nu A_{cs} }}$, and Womersley number based on hydraulic diameter. For Reynolds number, *Q*_*sys*_ is the temporal maximum of the local flow at each axial interval along the spine obtained by interpolation from the experimental data and ν is the kinematic viscosity of the fluid. Similar to previous studies, CSF viscosity was assumed to be that of water at body temperature. To evaluate the presence of laminar flow, (*Re* < 2300), similar to previous studies in CSF and biofluids mechanics, Reynolds number was evaluated at peak systolic flow along the spine. Womersley number, $\alpha = \frac{{D_{h} }}{2}\sqrt {\omega /\nu }$, where ω is the angular velocity of the volume flow waveform *ω* = 2*π*/*T*, was used to quantify the ratio of unsteady inertial forces to viscous forces. This ratio was previously found to be large relative to viscous forces by Loth et al. [29]. A value greater than 5 for Womersley number indicates transition from parabolic to “m-shaped” velocity profiles for oscillatory flows [30]. CSF pulse wave velocity (PWV) was quantified as an indicator of CSF space compliance. Timing of peak systolic CSF flow rate along the spine was determined based on our previously published method [31]. In brief, a linear fit was computed based on the peak systolic flow rate arrival time with the slope being equivalent to the PWV.

## Results

**Fig. 5:**
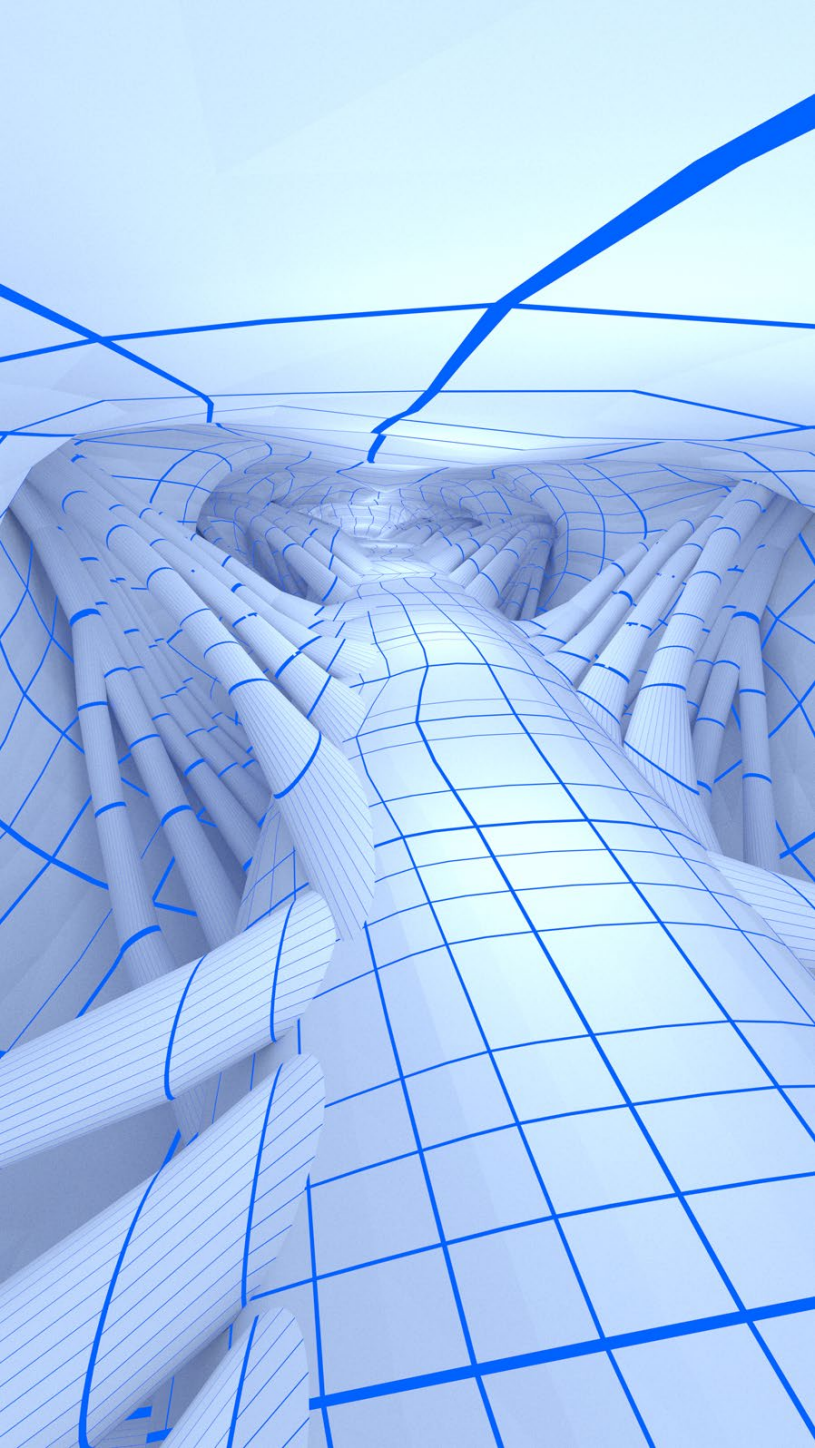
Visualization of the final quadrilateral surface mesh showing internal view of the spinal cord NR in the cervical spine with view in the caudal direction

The final model includes the 31 pairs of dorsal and ventral NR, spinal cord with coccygeal nerve and dural wall (Fig. 4). Final values for the vertical location where the NR join into the dura (Z position), radicular line, descending angle, root thickness, and number of rootlets for both dorsal and ventral NR are provided (Table 1). The percent difference of the final remeshed dura volume compared to the original dura segmentation was 2.7% (original segmentation volume = 100.5 cm^3^ and a final remeshed volume = 103.2 cm^3^). Addition of NR reduced the final remeshed volume to 97.3 cm^3^. A 3D visualization of the internal geometry is shown in Fig. 5.

**Table 1:** Anatomic measurements obtained from the final 3D spine model

		Dorsal rootlet measurements (average of left and right)				Ventral rootlet measurements (average of left and right)			
Nerve root #	Z position (mm from FM)	Descending angle (º)	Radicular line (mm)	Diameter (mm)	Number of rootlets	Descending angle (º)	Radicular line (mm)	Diameter (mm)	Number of rootlets
C1	6.1	6.8	10.1	0.7	4	− 7.8	8.3	0.7	3
C2	19.8	19.4	10.6	0.7	4	− 8.5	11.1	0.7	4
C3	38.9	45.2	15.5	0.7	6	28.3	13.7	0.7	5
C4	55.3	42.9	17.1	0.8	5	37.0	13.3	0.8	5
C5	71.1	47.5	13.9	0.8	5	37.3	10.7	0.8	5
C6	87.3	51.9	12.6	0.9	4	39.5	12.8	0.9	4
C7	105.4	54.8	11.5	1.0	4	59.6	4.9	1.0	4
C8	118.3	68.9	7.4	0.9	2	58.6	5.2	0.9	2
T1	132.3	70.2	6.1	1.1	3	64.3	7.5	1.1	3
T2	148.7	72.3	7.2	1.1	2	69.9	6.0	1.1	2
T3	169.2	70.4	7.1	1.1	2	68.6	7.2	1.1	2
T4	187.8	69.2	8.0	1.1	2	65.8	6.1	1.1	2
T5	209.9	73.5	10.5	1.1	2	73.1	9.7	1.1	2
T6	230.5	76.8	8.1	1.1	2	76.2	6.4	1.1	2
T7	258.0	78.7	8.2	1.1	2	78.7	8.5	1.1	2
T8	277.2	79.8	8.0	1.1	2	79.8	10.5	1.1	2
T9	307.5	79.7	8.3	1.1	2	79.7	6.1	1.1	2
T10	333.3	77.7	8.5	1.1	2	77.7	8.3	1.1	2
T11	366.2	77.4	8.5	1.1	2	77.4	6.5	1.1	2
T12	401.6	74.8	10.6	1.1	1	74.8	6.9	1.1	1
L1	436.3	82.7	1.1	1.1	1	82.6	1.1	1.1	1
L2	474.9	84.7	1.1	1.1	1	85.1	1.1	1.1	1
L3	510.7	87.1	1.5	1.5	1	88.0	1.5	1.5	1
L4	543.9	87.0	1.3	1.3	1	87.6	1.3	1.3	1
L5	570.6	87.3	1.2	1.2	1	86.9	1.2	1.2	1
S1	585.5	86.9	1.0	1.0	1	87.0	1.0	1.0	1
S2	595.3	86.6	0.5	0.5	1	87.5	0.5	0.5	1
S3	599.4	86.6	0.5	0.5	1	87.0	0.5	0.5	1
S4	602.0	86.8	0.5	0.5	1	87.3	0.5	0.5	1
S5	602.6	87.3	0.5	0.5	1	87.5	0.5	0.5	1

### Geometric parameters

Total intrathecal CSF volume below the foramen magnum was 97.3 cm^3^ (Table 3). Volumes of the dura mater, spinal cord and 31 NR pairs were 123.0, 19.9 and 5.8 cm^3^ respectively. The surface areas for the dura mater, spinal cord and NR were 318.5, 112.2 and 232.1 cm^2^ respectively. The average cross-sectional areas of the dura mater, spinal cord and NR were 2.03, 0.33 and 0.10 cm^2^ respectively. The length of the spinal cord down to the conus and spinal dura mater were ~44.8 cm and 60.4 cm respectively. Note, geometric parameters for the spinal cord were computed based on the spinal cord with the coccygeal nerve included as one continuous structure.

## 3D model files

Both quadrilateral and triangulated meshes for NR, spinal cord, and dura are provided (six files in total) with Creative Commons Attribution-ShareAlike 4.0 International (CC BY-SA 4.0) license (Additional file 1, note: file units are in millimeters). The number of polygons in the quadrilateral meshes of the NR, spinal cord and dura wall was 61,749, 35,905 and 27,281 respectively for a total of 124,935 quadrangles. The number of polygons in the triangulated meshes of the NR, spinal cord, and dura were 199,372, 71,870 and 54,613 respectively for a total of 325,855 triangles. In addition, to allow reduced order modeling of intrathecal CSF flow [32], a 1D graph of model x, y, z-coordinates for the dura and spinal cord centroids are provided in a Additional file 1. This file also contains the corresponding numeric values for all geometric and hydrodynamic parameters at 1 mm intervals along the spine.

### CSF flow

**Fig. 6:**
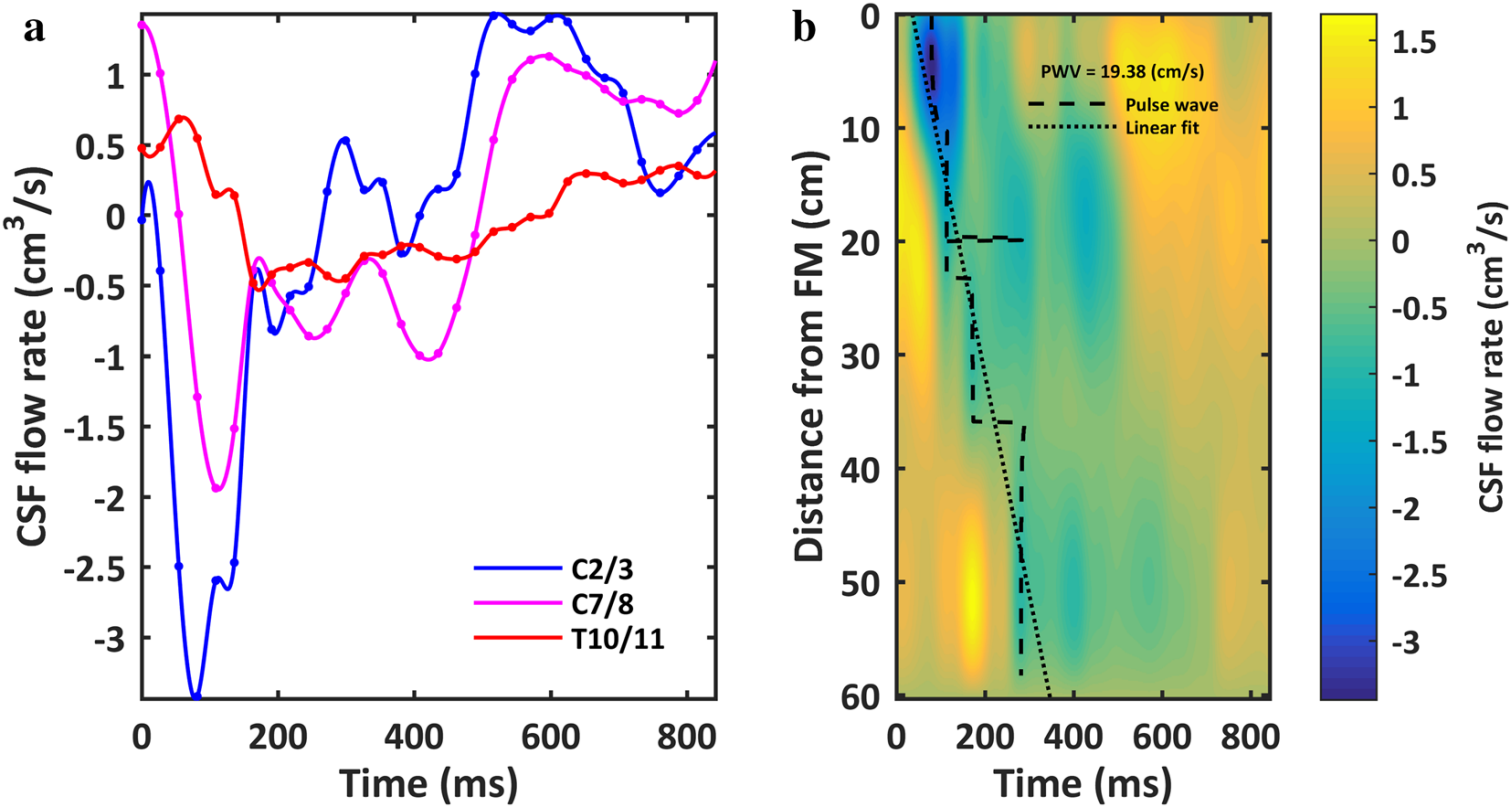
**a** Subject-specific CSF flow waveforms measured at C2/3, C7/T1 and T10/11 by phase contrast MRI. **b** Subject-specific quantification of CSF pulse wave velocity (PWV) along the spine estimated to be ~ 19.4 cm/s based on a linear fit (dotted line) of peak flow rate arrival times (dashed line)

**Fig. 7:**
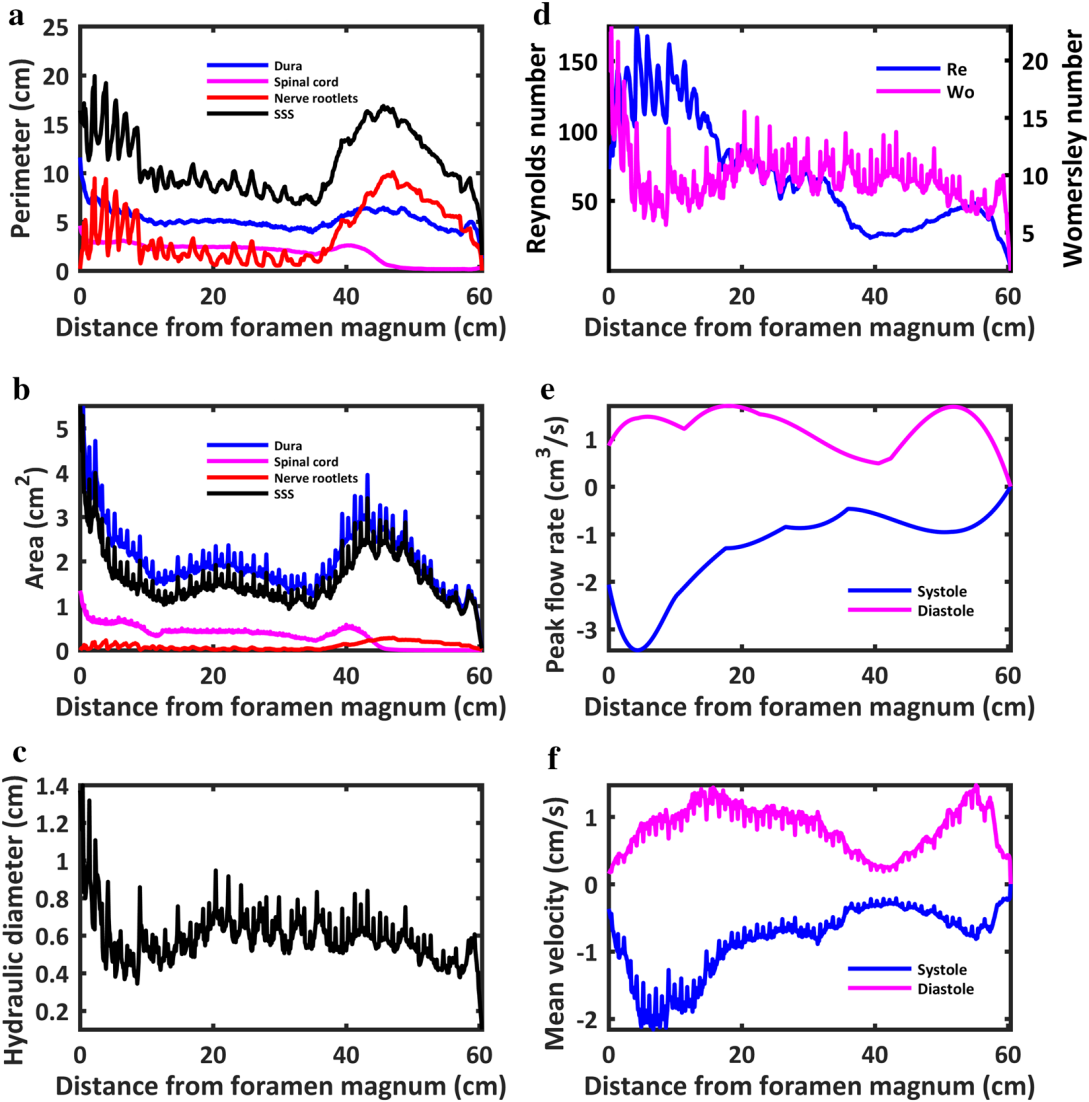
Quantification of axial distribution of geometric and hydrodynamic parameters in terms of **a** perimeter, **b** area, **c** hydraulic diameter, **d** Reynolds and Womersley number, **e** peak flow rate in the caudal direction (systole) and rostral direction (diastole), **f** mean velocity of CSF flow at peak systole and diastole

Peak-to-peak CSF flow amplitude measured at the C2–C3, C7–C8 and T10–T11 was 4.75, 3.05 and 1.26 cm^3^/s respectively (Fig. 6a). These were measured at an axial position relative to the model end (foramen magnum) of 4.0, 12.5, and 35.4 cm respectively. Based on the interpolated CSF flow waveform between MRI measurement locations, the maximum peak and mean CSF velocities were present at 38 mm (~ C4–C5, Fig. 7f). Minimum value of peak and mean CSF velocities occurred in the lower lumbar spine and within the thoracic spine from 390 to 410 mm (~ T7–T10, Fig. 7f).

Cerebrospinal fluid flow oscillation had a decreasing magnitude and considerable variation in waveform shape along the spine (Fig. 6a). Spatial temporal distribution of CSF flow rate along the SSS showed that maximum CSF flow rate occurred caudal to C3–C4 at ~ 40 mm (Fig. 6b). CSF pulse wave velocity (PWV) was estimated to be 19.4 cm/s (Fig. 6b).

### Hydrodynamic parameters

Average Reynolds and Womersley number was 68.5 and 9.6 respectively. Womersley number ranged from 1.6 to 22.96 (Table 2, Fig. 7d). Maximum Womersley number was present near the foramen magnum (*α* = 22.96). Womersley number had local minima within the cervical spine and just rostral to the intrathecal sac. Maximum Reynolds number was 174.9 and located at C3–C4.

**Table 2:** Summary of geometric and hydrodynamic parameters obtained from the final 3D spine model

Parameter	Average ± std.	Maximum	Minimum
Perimeter SC^a^ (cm)	1.85 ± 1.00	4.62	0.14
Perimeter DM (cm)	5.26 ± 1.01	11.59	1.25
Perimeter NR (cm)	3.84 ± 2.81	10.08	0.00
Perimeter SSS (cm)	10.96 ± 3.12	19.91	1.6
Area SC^a^ (cm^2^)	0.33 ± 0.23	1.34	0.00
Area DM (cm^2^)	2.03 ± 0.79	6.95	0.04
Area NR (cm^2^)	0.10 ± 0.08	0.28	0.00
Area SSS (cm^2^)	1.61 ± 0.65	5.62	0.04
Hydraulic diameter HD (cm)	0.59 ± 0.14	1.40	0.10
Re	68.46 ± 39.00	174.9	0.00
α	9.59 ± 2.27	22.96	1.64
U_sys_ (cm/s)	− 0.83 ± 0.51	N/A	− 2.16
U_dia_ (cm/s)	0.83 ± 0.34	1.47	N/A
Q_sys_ (cm^3^/s)	− 1.29 ± 0.87	N/A	− 3.44
Q_dia_ (cm^3^/s)	1.22 ± 0.39	1.69	N/A
PWV (cm/s)	19.4	N/A	N/A

^a^Average, maximum and minimum values are based 1 mm slice intervals along the entire spine including the coccygeal nerve

## Discussion

The intrathecal CSF space is a complex 3D fluid-filled geometry with multiple levels of anatomic complexity, the most salient features being the spinal cord, dura mater and dorsal and ventral spinal cord NR. An accurate anthropomorphic representation of these features is needed as a tool for development of in vitro and numerical models of CSF dynamics that can be used to inform and optimize CSF-based therapeutics. In this paper, we provide a detailed and downloadable anthropomorphic 3D model (Additional file 1) of the intrathecal CSF space that is licensed for re-use under the Creative Commons Attribution-ShareAlike 4.0 International license (CC BY-SA 4.0). CSF flow data, measured by PCMRI, is provided as a validation data set for numerical modeling. The model is characterized in terms of axial distribution of intrathecal CSF dynamics with detailed information on various hydrodynamic parameters including Reynolds number, Womersley number, hydraulic diameter and CSF velocities. Herein, we discuss the model in terms of its segmentation, remeshing, key modeling considerations and comparison to previous anatomic and modeling studies and in vivo CSF dynamics measurements.

### Segmentation of the intrathecal CSF space

A variety of software exists to help reconstruct MRI DICOM image files in 3D. Many segmentation software platforms provide automatic segmentation algorithms that can deliver relatively quick visualizations but these segmentations are often not suitable to create 3D models that can be used for CFD modeling or easily exported for 3D printing [33]. In this study, we used the open-source program ITK-SNAP (“The Insight Segmentation and Registration Toolkit”, http://www.itk.org) that supports automatic, semi-automatic and manual approaches. The final model was constructed based on manual segmentation of each slice along the spine by an expert operator previously trained in intrathecal CSF segmentation procedures.

Despite the popularity of CFD studies conducted in the SSS, there is a lack of detailed information on intrathecal segmentation methods based on anatomic MR imaging. The craniocervical junction is highly vascularized with relatively large blood vessels that transverse the region, including the vertebral arteries (3.7 mm diameter for the left vertebral artery and 3.4 mm diameter for the right vertebral artery [34]) and the anterior spinal artery (0.3–1.3 mm diameter [35]). Spinal cord NR can sometimes be seen as dark regions crossing the SSS (Fig. 1d–f). Their length and obliqueness increases progressively moving towards the feet [36]. Denticulate ligaments are located between adjacent sets of NR in the cervical and thoracic spinal cord segments. These structures are too small to be quantified by MRI (thickness of ~ 0.1 mm) but may also appear as slightly darkened regions of SSS on each side of the spinal cord. The CSF on the anterior or posterior side of the spinal cord near the foramen magnum may appear dark in coloration due to flow void artifacts resulting from elevated CSF velocities at this region (and others along the SSS, Fig. 1). Although these regions can appear relatively dark on MR imaging, they should be considered as fluid.

Along the entire spine, the epidural space can appear hyper intense due to the presence of epidural fat (Fig. 1e–f). Care should be taken to not confuse these areas with CSF as it can be difficult to visualize the relatively thin dura mater that separates the two spaces. This ambiguity often confounds automatic segmentation tools and thresholding should be reviewed in detail to ensure accuracy. From our experience, no presently available automated algorithm can avoid over-segmentation of epidural fat, as there can be virtually no border visible between these two regions at many locations along the spine due to MR image resolution limits that do not allow visualization of the relatively thin dura.

The cauda equina begins around the conus medullaris that is located near the lower border of the first lumbar vertebra. This structure is formed by the long rootlets of the lumbar, sacral and coccygeal nerves that run vertically downward to their exit. Similar to the spinal cord NR, ligaments and blood vessels, these small bundles of nerves are not possible to accurately quantify with the current MR image resolution through segmentation alone. In the presented model, they are modeled as curving cylinders as described in our methods with reference to cadaveric studies in the literature and visual interpretation and measurement of NR insertion at the spinal cord and dura.

### Modeling considerations with small anatomy

Although the spinal cord and dura mater were easily visible, smaller structures such as NR were not clearly discernible in the MRI scans used in this study. In our previous study [36], we grossly modeled spinal cord NR as single airfoil shaped structures within the cervical spine only. For the present complete spine model for a healthy subject, we individually modeled the number of nerve rootlets at all vertebral levels (see Fig. 4 for anatomic depiction of nerve rootlets and Table 1 for number of nerve rootlets). The nerve rootlets were each placed with reference to the high-resolution MR imaging, 3D segmented geometry and published cadaveric measurements and images in the literature. Because no single source contained enough information to fully reconstruct the NR geometry, the final model does not strictly adhere to any single set of tabular parameters, but rather, is a best judgment based on the collective information (see Table 1 for parameters). Furthermore, due to limitations in the data as well as the time intensive nature of the modeling process, NR were mirrored left to right along the spinal cord. The duplicate side was subjected to < 3.0 mm translation as necessary to best fit rootlets to the spinal and dural geometry. NR vertical positioning is only referenced by the corresponding vertebral level in the literature. Therefore, vertical positioning was based solely on segmentation data marking SSS entry and exits locations. The resulting model is subject-specific in terms of NR location and orientation, but idealized in terms of the exact structure (Fig. 4).

#### Volumetric differences in geometry

A large portion of this work is centered on the quadrilateral remeshing of the spinal and dural surfaces. In this case, introducing volumetric error was a primary concern during this process. This was largely compensated by selectively increasing mesh resolution in areas with higher degree of curvature while reducing resolution in locations with little curvature. However, discrepancies still occurred and it was necessary to further modify the entire surface fit as described in the “Methods”. Excluding the NR, which were not originally segmented, the final difference between segmented and remeshed SSS volumes is 2.7% (Fig. 3). Our previous study showed inter-operator volumetric error for SSS CSF segmentation to be < 2.7% [24], a value comparable to the percent difference in the remeshed volume for the present study. In an in vitro cervical SSS model, segmentation inaccuracy was quantified to be 15% larger than the original geometry STL file used to create the model [37]. In combination, these findings indicate a high-degree of segmentation and remeshing reliability, but do not rule out the possibility for significant degree of segmentation inaccuracy. Unfortunately, the true SSS geometry is not known and therefore not possible to validate for accuracy.

### Comparison of model CSF volume to measurements in the literature

**Fig. 8:**
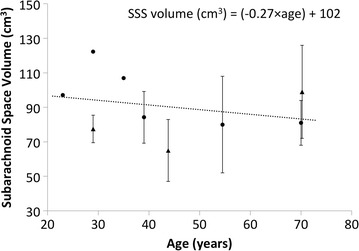
Summary of spinal subarachnoid space (SSS) volumes computed in published studies in the literature using MR imaging applied for adult-aged subjects (studies in Table 3). A decreasing trend in SSS CSF volume occurs with age (error bars represent standard deviations, triangles indicate studies with patients and circles indicate studies with healthy controls)

While the provided model is subject-specific, it can be compared to other MRI-based studies to help understand its similarity to the general population. Overall, the provided model had a SSS volume of 97.34 cm^3^ and showed a strong similarity with the previous studies cited that, on average, reported SSS volume to be 90.3 cm^3^ [38–45]. Table 3 gives a review of studies that used MRI to quantify the volume of anatomical features within the full spine and lumbosacral spine for healthy subjects. In collection, these published studies indicate a decreasing trend in CSF volume with age given by: *SSS*_*volume*(*ml*)_ = ( − 0.27 × *age*) + 102 (Fig. 8). The provided model had a volume that was on the higher end of the average reported values, however it was also for a relatively young 23-year-old subject (Table 3). It should be noted that the model was based on high-resolution 0.5 mm isotropic MR images, whereas all cited studies were based on MR images with considerably lower resolution. In addition, many of these studies used axial images with ~ 8 mm slice spacing and a relatively large slice thickness.

**Table 3:** Review of studies that include volumetric quantification of anatomic regions within the spine using MR imaging

Source	Region	SC (cm^3^)	NR (cm^3^)	SSS (cm^3^)	N	Mean age	Segmentation method	Subject type
Current study (Sass et al. (2017))	Full spine	19.9	5.8	97.3	1	23	Manual	Healthy
Hogan et al. [38]	Full spine	–	–	107^a^	2	35	Manual	Healthy
Edsbagge et al. [39]	Full spine	20	–	81 ± 13	22	70	Semi-automated	Healthy elderly
Bagci et al. [43]	Full spine	–	–	86	1	–	Automated	Idiopathic intracranial hypertension
Hsu et al. [40]	Full spine	–	–	122	1	29	Manual	Healthy
Lebret et al. [44]	Full spine	–	–	99 ± 27	6	70.2	Automated	Non-communicating hydrocephalus
Lebret et al. [44]	Full spine			80 ± 28	12	54.5^b^	Automated	Healthy
Lebret et al. [44]	Full spine	–	–	65 ± 18	20	43.8^b^	Automated	Communicating hydrocephalus
Alperin et al. [41]	Full spine	21.0	–	78 ± 8	8	29	Automated	Obese women only
Levi Chazen et al. [42]	Full spine	–	–	84 ± 15	15	39	Automated	Healthy
Current study (Sass et al. (2017))	Lumbosacral	3.8	4.0	51.0	1	23	Manual	
Hogan et al. [38]	Lumbosacral	–	7.31	49.9	25	35	Manual	
Carpenter et al. [47]	Lumbosacral	–	–	53.7^a^	41	33	Manual	
Higuchi et al. [48]	Lumbosacral	–	–	41.7^a^	41	30	Manual	
Sullivan et al. [45]	Lumbosacral	–	–	35.8	71	48	Automated	
Edsbagge et al. [39]	Lumbosacral	–	13	25	22	70	Semi-automated	
Martyr et al. [46]	Lumbosacral	–	9.2	31.8	16	72	Automated	
Puigdellivol et al. [49] , [87]	Lumbosacral	–	–	34.4	7	37	Semi-automated	
Prats Galino et al. [50]	Lumbosacral	–	10.4	34.3	7	38	Semi-automated	
Hsu et al. [40]	Lumbosacral	–	–	53.0	1	29	Manual	

^a^Indicates studies where NR volume was included in the calculation

^b^Value obtained from personal correspondence with author

The provided subject-specific 3D model was based on a combination of subject-specific MR imaging (Fig. 1) and cadaveric measurements by Bozkurt et al. [25], Zhou et al. [26], Hauck et al. [27] and Lang et al. [28]. The cadaveric studies used to define the NR specifications were selected based on their completeness of information that included spinal cord NR descending angle, radicular line and diameter. As expected, a local enlargement of the spinal cord cross-sectional area was present near the lumbosacral (L2–S2) and cervical (C5–T1) enlargements located near 13 and 40 cm respectively below the foramen magnum (Fig. 7). These locations corresponded to the expected enlargement due to gray matter increase within those regions.

The exact 3D structure of the 31 NR pairs and coccygeal nerve were idealized based on the literature as it was not possible to extract their exact detailed geometry directly from MR imaging. However, it was possible to place each NR pair on a subject-specific basis at the insertion point in the spinal cord and exit point at the dura (details in “Methods”). The resulting model had a total NR volume of 5.8 cm^3^. This value is similar to that quantified by Hogan et al. (1996) and Martyr et al. (2011) with 7.31 and 9.2 cm^3^ respectively [38, 46]. The relatively smaller volume in our model is likely due to the smaller size of NR between the L2–S2 levels in comparison to Hogan’s cadaveric measurements [40]. In addition to the noted wide individual variability, Hogan et al. [38] estimated NR volume assuming estimate root lengths from relatively low resolution MRI data. Other studies quantifying cauda equina volume also based their results solely on estimations from MRI segmentations [39, 45–50].

### Total CSF volume in healthy adults

Total CSF volume in healthy adults has been reported to be ~ 150 mL in many standard medical textbooks [42, 51, 52] and recently published review articles [53, 54]. This value has become ubiquitous within the literature to the point of often not being cited with reference to any empirical study. Methods for CSF volume estimation by relatively crude casting techniques were originally applied [55]. These estimates were later criticized as being prone to significant degree of error [56, 57]. Review of more recent literature using non-invasive MRI-based methods indicates that total CSF volume in healthy adults to range from ~ 250 to 400 cm^3^ [42, 58–61]. The difference in CSF volume determined from MRI versus invasive techniques is likely an underlying reason for the discrepancy. The referenced CSF volumetric studies using non-invasive techniques with high-resolution MR imaging may provide a more accurate estimate of total CSF volume. However, invasive measurements provide a lower bound for total CSF volume. More research is needed to fully establish detailed information about CSF volumetric distribution throughout the intracranial cisterns and subarachnoid space of the brain and spine.

### Comparison of 3D model with previous geometries used for CFD modeling

At present, all models of the spinal SSS rely on varying degrees of simplification or idealization, often neglecting realistic spinal canal geometry and/or microanatomy. The simplest geometries are coaxial circular annuli employed by Lockey et al. [62], Berkouk et al. [63], Hettiarachchi et al. [64] and Elliott [65] that in some cases also included pathological variations, as well as in Bertram et al. [17] which used an idealized axial distribution for SSS area. Stockman [66] used an elliptical annuli and included microanatomical features, whereas Kuttler [67] modeled an elliptical annulus based on work by Loth et al. [29] who created a SSS from realistic SSS cross sections. The axial distribution of our model spinal cord and dura shows strong similarity to Loth et al. [29], Fig. 3, with a peak SSS area located at the FM and dural sac lumbar enlargement (Fig. 7b). Hsu et al. [40], Pahlavian et al. [36] and Tangen et al. [10, 12] developed CFD models with a subject specific geometry of the SSS reconstructed from MR data. The Pahlavian and Tangen CFD models also included varying degrees of NR detail. Pahlavian idealized NR as smooth airfoil-shaped flat objects and limited the model to the cervical spine. Yiallourou et al. [68] conducted a CFD study to investigate alterations in craniocervical CSF hydrodynamics in healthy controls versus patients with Chiari malformation. In that study, NR were not included in the CFD geometry. The CFD-based velocity profile results were found to lack similarity with in vivo 4D Flow MRI measurements. It was concluded that NR or other relatively small anatomic features are likely needed to accurately reflect CSF velocities within the cervical spine.

The geometric model presented in this study contributes NR microanatomy as discreet rootlets and cauda equina within a complete subject-specific SSS geometry. The model geometry is provided in a downloadable format with the dura, spinal cord and NR as separate files in the .STL (triangular) and .OBJ (quadrilateral) formats (six files in total). This allows modification of each surface separately for modeling purposes. For example, the model could be altered locally to increase the thecal sac volume during upright posture.

### CSF dynamics quantification

The computed parameters for CSF dynamics in terms peak flow rate, mean velocity and Reynolds number (Fig. 7) compare favorably to previous studies. The measured CSF flow rate waveforms (Fig. 6a) had similar magnitude as previous studies in the literature by Loth et al. [29], Linninger et al. [69] and Greitz [70, 71]. For those studies, average value of the peak CSF velocity at C2 vertebral level was ~ 2.5 cm/s. In the present model, peak CSF velocity at C2 vertebral level was 2.16 cm/s (Fig. 7f, towards feet). CSF pulse wave velocity (PWV), was estimated to be 19.4 cm/s in the healthy subject based on feature points of the CSF flow waveform measured along the entire spine (Fig. 6b). This value is lower than those previously reported in the literature that include 4.6 ± 1.7 m/s by Kalata et al. in the cervical spine [31] and ~ 40 m/s by Greitz in a patient [72]. It is difficult to directly compare these results with the present study, as they varied in technique, measurement location and type of subject.

Peak Reynolds number was predicted to be 175 and located within the cervical spine. This value suggests the presence of laminar CSF flow throughout the intrathecal space. However, it should be noted that that the SSS is a highly complex geometry that also contains microscopic structures called arachnoid trabeculae that were not included in the flow calculations. Previous biofluids studies have shown that geometric complexity can allow flow to become partially turbulent at Re > 600 in a stenosis [73], at Re 200–350 in aneurysms [74, 75], in the heart [76] and within CSF in the SSS [77, 78]. More research is needed to define the nature of CSF flow dynamics with respect to turbulence.

Cerebrospinal fluid flow data was collected at three distinct axial locations along the spine for a single subject. Data from these three locations was spatial-temporally interpolated (Fig. 6b) and used in combination with the geometry to quantify axial distribution of CSF dynamics along the spine (Fig. 7). While only representative of the single subject analyzed, the provided parameters give insight into CSF dynamics for a single healthy subject within a complete SC model containing detailed nerve root geometry. For example, the detailed geometry showed that Reynolds number varies significantly along the spine due to the presence of NR (see Fig. 7d Reynolds number variation in cervical spine). Note: validation of numerical models using the provided downloadable CSF flow waveform data should only take into account CSF flow rates measured at the three distinct axial locations (Fig. 6a). Interpolated values are not empirical data to be used for validation purposes.

## Limitations

The provided anthropomorphic model of intrathecal CSF has several important limitations. Our model included the dorsal and ventral spinal cord NR with semi-idealized geometry that was mirrored across the spinal cord for a healthy subject. For a diseased case, such as in patients with syringomyelia or Chiari malformation, it is expected that the exact NR position may be altered. In the case of syringomyelia, the SSS has been found to narrow near the syrinx [79] and would likely result in local displacement of NR towards the dura. The present model may not be relevant for representing such a diseased case.

We sought to render the NR structures as near as possible to reality based on a combination of referencing the in vivo MR imaging and cadaveric measurements in the literature. However, the resulting model cannot be considered truly subject-specific, as the exact locations and geometry of each NR was not possible to directly visualize. Higher resolution MRI would be required to construct such a model. In addition, several additional anatomic features are missing in the model including: denticulate ligaments and tiny blood vessels that transverse the intrathecal CSF spaces. Additional work could be made to add these features to the model in an idealized way.

The provided model only includes CSF within the intrathecal space. This was due to MRI scanning time limitations. The protocol used in the present study required 45 min of scanning time to obtain the necessary high-resolution complete spine imaging. Future studies should quantify the entire CSF space geometry in detail to allow modeling of Chiari malformation and other intracranial central nervous system diseases.

Cerebrospinal fluid flow data used for calculation of CSF dynamics along the spine was measured at three axial positions along the spine. An improved method would include measurement of CSF flow at more axial levels and with higher temporal resolution. The exact reproducibility of these CSF flow waveforms could be tested by conducting a reliability study on the same subject. In this study, cardiac-related CSF flow was quantified using retrospective gated PCMRI measurements. Therefore, Fig. 7 results indicate CSF hydrodynamics under cardiac-related CSF oscillations. Impact of the respiratory cycle on CSF flow dynamics could be quantified using real-time PCMRI [80–83].

## Conclusions

This study provides an anatomically realistic anthropomorphic 3D model of the complete intrathecal space based on high-resolution MR imaging of a healthy human adult female. The axial distribution of CSF dynamics within the model are quantified in terms of key hydrodynamic and geometric variables and likely indicate laminar CSF flow throughout the SSS. The model (Additional file 1) is provided for re-use under the Creative Commons Attribution-ShareAlike 4.0 International license (CC BY-SA 4.0) and can be used as a tool for development of in vitro and numerical models of CSF dynamics for design and optimization of intrathecal drug delivery, CSF filtration, CSF hypothermia and central nervous system diseases of the SC such as syringomyelia and spinal arachnoiditis.

## Additional file

**Additional file 1. **3D computer aided design files for the spinal cord, dura and nerve roots in .OBJ and .STL file format.  Summary of geometric and hydrodynamic results for the spinal cord, dura and nerve roots in .XLSX format.

## Abbreviations

3D: three-dimensional
ASA: anterior spinal artery
CFD: computational fluid dynamics
CSF: cerebrospinal fluid
DM: dura mater
DA: descending angle
ES: epidural space
FIESTA: fast imaging employing steady-state acquisition
FM: foramen magnum
FOV: field of view
LV: left vertebral artery
MR: magnetic resonance
MRI: magnetic resonance imaging
NR: nerve rootlets
PWV: pulse wave velocity
RL: radicular line
RV: right vertebral artery
SC: spinal cord
SSS: spinal subarachnoid space
TE: echo time
TR: repetition time
